# Tissue-Specific Mechanism of Fat Distribution in Teleosts: Comparative Analysis Between Two Carnivorous Marine Species, Golden Pompano (*Trachinotus ovatus*) and Spotted Sea Bass (*Lateolabrax maculatus*)

**DOI:** 10.1155/anu/1005455

**Published:** 2025-11-03

**Authors:** Ningning Su, Jun Zheng, Guanrong Zhang, Wenqiang An, Fang Chen, Chao Xu, Dizhi Xie, Yuanyou Li

**Affiliations:** ^1^College of Marine Sciences, South China Agricultural University, Guangzhou 510642, Guangdong, China; ^2^College of Animal Science and Technology, Henan Agricultural University, Zhengzhou 450046, China

**Keywords:** adipogenesis, *Lateolabrax maculatus*, lipid metabolism, tissue specificity, *Trachinotus ovatus*

## Abstract

The molecular mechanisms underlying species-specific lipid distribution patterns in teleosts remain poorly understood. This study investigated two marine carnivorous species with distinct fat deposition characteristics: the golden pompano (*Trachinotus ovatus*), which stores lipid in the liver and muscle, and the spotted sea bass (*Lateolabrax maculatus*), which primarily stores fat in the abdominal adipose tissue. Juveniles (~10.0 g) were fed three iso-nitrogenous diets (45% protein) with graded lipid levels (12%, 14%, and 16%) for 8 weeks. Two-way analysis of variance (ANOVA) analyses revealed significant species × diet interactions affecting lipid distribution (*p* < 0.05). Golden pompano exhibited higher hepatic/muscular crude lipid and triglyceride (TG) levels than spotted sea bass, whereas abdominal TG content and abdominal fat index (AFI) were lower (*p* < 0.05). Transcriptomics and qPCR revealed tissue-specific regulatory mechanisms: there was an upregulation of hepatic and muscular fatty acid transport genes (*ldlr* and *fabp*), synthesis (*g6pd*), and deposition (*dgat1*) with increasing dietary lipid in golden pompano. Additionally, enhanced adipogenesis (*c/ebpα* and *pparγ*) and TG storage (*dgat1*) were observed in the abdominal adipose of spotted sea bass. These findings indicate that lipid accumulation in the liver and muscle of golden pompano is driven by increased fatty acid transport and lipogenesis, while spotted sea bass prioritizes abdominal adipogenesis. This study provides novel insights into the regulation of lipid metabolism in teleosts, with implications for aquaculture feed optimization.

## 1. Introduction

Lipid distribution in teleosts is a critical determinant of both aquaculture efficiency and consumer preference. Unlike mammals, fish exhibit remarkable interspecific diversity in lipid storage patterns. Species such as largemouth bass (*Micropterus salmoides*) predominantly store lipids in abdominal adipose tissue [1], while Atlantic cod (*Gadus morhua*) and obscure puffer (*Takifugu obscurus*) prioritize hepatic lipid deposition [2, 3]. These differences are not merely anatomical but reflect evolutionary adaptations to ecological niches and energy utilization strategies. For instance, migratory species such as Atlantic salmon (*Salmo salar*) and triploid rainbow trout (*Oncorhynchus mykiss*) mainly store lipids in their fillets to sustain energy during long-distance movements [4, 5]. Such interspecific variations in lipid partitioning influence fillet nutritional profiles and may affect disease susceptibility through ectopic lipid accumulation. Current research has primarily focused on characterizing lipid distribution phenotypes through histological analyses, lipidomic profiling, and physiological assessments of lipid mobilization [1, 6–9]. However, the underlying mechanism of lipid distribution in different tissues remains unknown. Elucidating these molecular mechanisms could provide novel insights for improving fish health, fillet quality, and product yield rates.

Tissue-specific lipid deposition in fish is regulated through fatty acid metabolism, which encompasses several core processes: fatty acid uptake, β-oxidation, de novo lipogenesis, intracellular reesterification, and adipose tissue expansion [10, 11]. The coordination of these metabolic pathways depends on a network of rate-limiting enzymes and transcriptional regulators. Lipoprotein lipase (*lpl*) hydrolyzes the triglycerides (TG) in circulating lipoproteins into free fatty acids (FFAs) and lipoprotein remnants [12]. Subsequently, FFAs are internalized via the transmembrane transporter cluster of differentiation 36 (*cd36*) and transported intracellularly by fatty acid binding proteins (*fabps*), which determine their metabolic fates—either storage as cytoplasmic lipid droplets (TG synthesis) or mitochondrial β-oxidation for energy production [13, 14]. In addition to dietary uptake, fatty acids derived from de novo lipogenesis contribute significantly to lipid deposition [15]. Key enzymes in this pathway, including fatty acid synthesis (*fas*) and glucose-6-phosphate dehydrogenase (*g6pd*), are transcriptionally suppressed in the liver of fish under high-lipid dietary conditions, as demonstrated in European seabass (*Dicentrarchus labrax*) [16, 17]. Intracellular reesterification—the conversion of noncatabolized FFAs into TG—represents another critical mechanism for lipid retention in peripheral tissues. Experimental evidence from murine models indicates that muscle-specific overexpression of diacylglycerol acyltransferase 1 (*dgat1*), a rate-limiting enzymes in TG synthesis, promotes ectopic lipid accumulation [18, 19]. Furthermore, lipid deposition is modulated by adipocyte differentiation dynamics. Master transcriptional regulators such as peroxisome proliferator-activated receptor gamma (*pparγ*) and CCAAT/enhancer binding proteins (*c/ebps*) can alter the distribution of body lipid through adipose tissue expansion in specific depots [20, 21].

Golden pompano (*Trachinotus ovatus*) and spotted sea bass (*Lateolabrax maculatus*) are commercially important marine carnivorous species that represent two distinct lipid storage patterns, making them ideal models for investigating species-specific mechanisms of lipid partitioning. Recent studies have explored the nutritional requirements of these species in terms of protein, lipid, and other essential nutrients. The optimal dietary protein levels range from 42% to 49% for golden pompano and from 41% to 47% for spotted sea bass, while the appropriate dietary lipid level is 12% for both fish species [22–25]. Nevertheless, the mechanisms underlying lipid distribution in these two species remain poorly understood. Therefore, this study aimed to preliminarily explore the molecular basis of lipid distribution patterns by analyzing growth performance, body fat distribution, transcriptome profiles, and the expression levels of lipid metabolism-related genes in the muscle, liver, and abdominal adipose tissue of golden pompano and spotted sea bass under varying dietary lipid conditions.

## 2. Materials and Methods

### 2.1. Experimental Animals and Procedures

The experimental processes were approved by the Institutional Animal Care and Use Committee of South China Agricultural University. Juvenile golden pompano and spotted sea bass were obtained from a local fish hatchery. To facilitate acclimation to the experimental diets and conditions, the fish were initially fed a combination of commercial feed and equal portions of diets L12, L14, and L16 for 15 days in floating net cages (1.0 m × 1.0 m × 1.5 m) before the formal trial. After acclimation, 270 healthy juveniles of each species were randomly distributed into nine net cages per species. Each dietary group consisted of three cages, with 30 fish per cage. The net cages for the two species were separated and arranged on the same wooden fishing raft. The initial body weight of the fish was recorded. The feeding trial lasted 8 weeks and was conducted simultaneously in the same sea area. Fish were fed to apparent satiation twice daily at 7:00 a.m. and 5:30 p.m. under natural light conditions. Water quality parameters, including salinity (32–33‰), temperature (25–28°C), dissolved oxygen levels (>5.0 mg L^−1^), and ammonium nitrogen (NH4^+^-N) concentrations (<0.5 mg L^−1^), were monitored twice weekly.

### 2.2. Experimental Diets

Three iso-nitrogenous experimental diets (45% crude protein) were formulated with graded lipid levels: 12% (L12), 14% (L14), and 16% (L16). A blended oil was used as the lipid source. The detailed ingredient composition and proximate nutritional analysis are presented in Table 1. All ingredients were sieved through a 60-mesh screen (250 µm) and thoroughly mixed with oil and distilled water using a Hobart-type mixer to ensure homogeneity. The mixture was then extruded into 2 mm in diameter pellets using an automatic pellet-making machine (SLC-45, Fishery Machinery and Instrument Research Institute, China). The pellets were air-dried to approximately 10% moisture, vacuum-sealed, and stored at −20°C until use.

### 2.3. Sample Collection

Before sampling, fish were fasted for 24 h. All fish from each cage were weighed and counted. Six fish per cage were randomly selected, anesthetized with MS-222 (Sigma, USA), and dissected to collect blood, liver, muscle, and abdominal adipose tissue. The liver, visceral tissue, and abdominal adipose tissue were weighted to calculate the hepatosomatic index (HSI), viscerosomatic index (VSI), and abdominal fat index (AFI). Blood samples were collected from the tail vein using sterile 1 mL syringes, allowed to clot at 4°C for 1–4 h, and centrifuged at 3000 *g* for 15 min at 4°C to obtain serum. The serum was aliquoted and stored at −80°C for subsequent biochemical analysis. Tissue samples, including muscle, liver, and adipose tissue, were flash-frozen in liquid nitrogen and stored at -80°C for further analysis.

### 2.4. Serum Biochemical Parameters

Serum concentrations and activities of TG, high-density lipoprotein (HDL), cholesterol (CHOL), nonesterified fatty acid (NEFA), low-density lipoprotein (LDL), and alkaline phosphatase (ALP) were quantified using a multimode reader (BioTek Instruments, Inc., Vermont, USA). All measurements were conducted with commercially available assay kits (Nanjing Jiancheng Co., Nanjing, China) according to the manufacturer's instructions.

### 2.5. Transcriptome Sequencing and Analysis

Total RNA was extracted from liver and muscle tissues using TRIzol reagent (Invitrogen, Carlsbad, USA). RNA integrity and quantitation were assessed using agarose gel electrophoresis and a NanoDrop ND-2000 spectrophotometer. RNA-seq libraries were constructed from 1 μg high-quality total RNA (RIN ≥8.0, assessed by Agilent 2100 Bioanalyzer) using the Illumina TruSeq Stranded mRNA Library Prep Kit (San Diego, CA, USA). For the analysis of differential expression of genes (DEGs) between the L12 and L16 groups in the liver and muscle of golden pompano and spotted sea bass, transcript quantification employed the transcripts per million (TPM) normalization method, with differential expression analysis conducted through DESeq2's negative binomial generalized linear model. Gene abundances, differential expression, and KEGG pathway analysis were performed using RSEM [26], DESeq2 [27], and KOBAS [28], respectively.

### 2.6. qRT-PCR Analysis

Total RNA isolation from liver, muscle, and adipose tissues using AG RNAex Pro Reagent (Accurate Biotechnology Co., Ltd., China) according to the manufacture's instruction. RNA integrity and concentration were assessed using the NanoDrop 2000 spectrophotometer and 1.2% agarose gel visualization. Reverse transcription was conducted with 1 μg total RNA using the PrimeScript RT Master Mix (Takara, Japan), incorporating genomic DNA eraser treatment. Gene-specific primers were designed using Primer Premier 5.0 Software, based on the genome database (Accession numbers: GCA_031216445.1 and GCA_036281595.1) and unpublished transcriptome data of golden pompano and spotted sea bass. The primer sequences are presented in Table 2. Prior to RT-qPCR analysis, primer specificity and amplification efficiency were validated through standard curve analysis with serially diluted cDNA. The quantitative PCR was amplified in a CFX Connect real-time PCR system (Bio-Rad, Singapore) using SYBR qPCR Mix (Toyobo Co., Ltd.) in 10 μL reactions, comprising 5 μL master mix, 1.0 μL cDNA template, 0.5 μL each of forward and reverse primers (10 μM), and 3 μL DNase/RNase-free water. The real-time PCR conditions were as follows: 95°C for 30 s, 40 cycles at 95°C for 5 s, and annealing at 60°C for 30 s. The *β-actin* gene served as the housekeeping gene. Relative quantification used the 2^−ΔΔCt^ method [29] with efficiency correction (90%–110% via five-point standard curve; *R*^2^ > 0.99).

### 2.7. Statistical Analysis

All experimental data are expressed as mean ± standard error. Statistical analyses were performed using SPSS 25.0 (SPSS Inc., Chicago, IL). One-way analysis of variance (ANOVA) followed by Tukey's test was employed to statistically evaluate multiple comparisons among different dietary lipid levels for the same fish species. Differences between the two species at the same lipid level were evaluated using an independent samples *t*-test. Two-way ANOVA was used to evaluate the main effects (dietary lipid levels and fish species) and their interactions. Statistical significance was defined at *p* < 0.05. All ANOVA assumptions were verified prior to analysis.

## 3. Results

### 3.1. Growth Performance and Morphological Index

**Figure 1:**
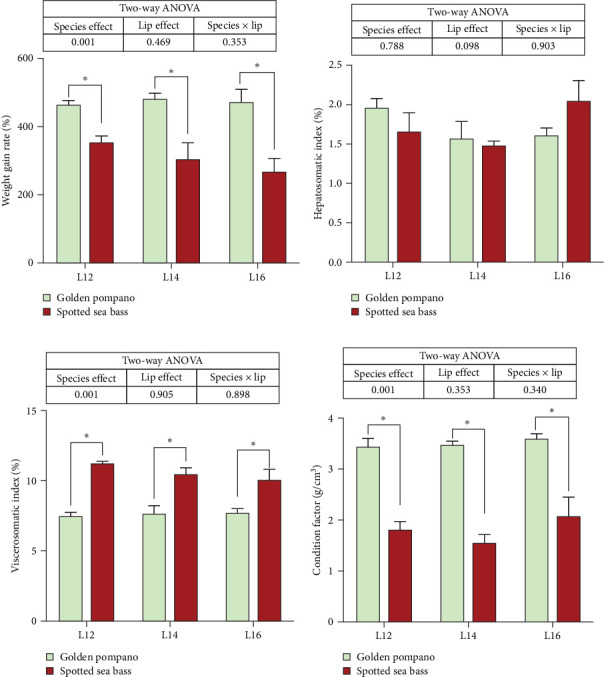
(a–d) Comparison of growth performance and morphological index of gold pompanos and spotted sea bass after feeding with the experimental diets for 8 weeks. The difference between golden pompano and spotted sea bass fed the same diet was compared using *t*-test (*⁣*^*∗*^*p* < 0.05). Values were analyzed by two-way ANOVA (factors: species × dietary lipid levels). Main effects and interactions with *p* < 0.05 were considered statistically significant. Data are presented as mean ± SEM (*n* = 3 replicates). WGR (weight gain rate, %) = 100 × (final body weight – initial body weight)/initial body weight; HSI (hepatosomatic index, %) = 100 × liver wet weight/body wet weight; VSI (viscerosomatic index, %) = 100 × visceral wet weight/body wet weight; CF (condition factor, g/cm^3^) = 100 × weight/body length.

Statistical analysis revealed that the interaction between fish species and dietary lipid levels showed no significant effect on weight gain rate (WGR), HSI, VSI and condition factor (CF) (*p* > 0.05), as depicted in Figure 1. When examining the main effects separately, dietary lipid levels showed no significant impact on WGR, HSI, VSI and CF (*p* > 0.05). However, VSI displayed significant differences between the two fish species (*p* < 0.05).

### 3.2. Lipid Content and TG Concentration in the Liver, Muscle, and Adipose Tissue

**Figure 2:**
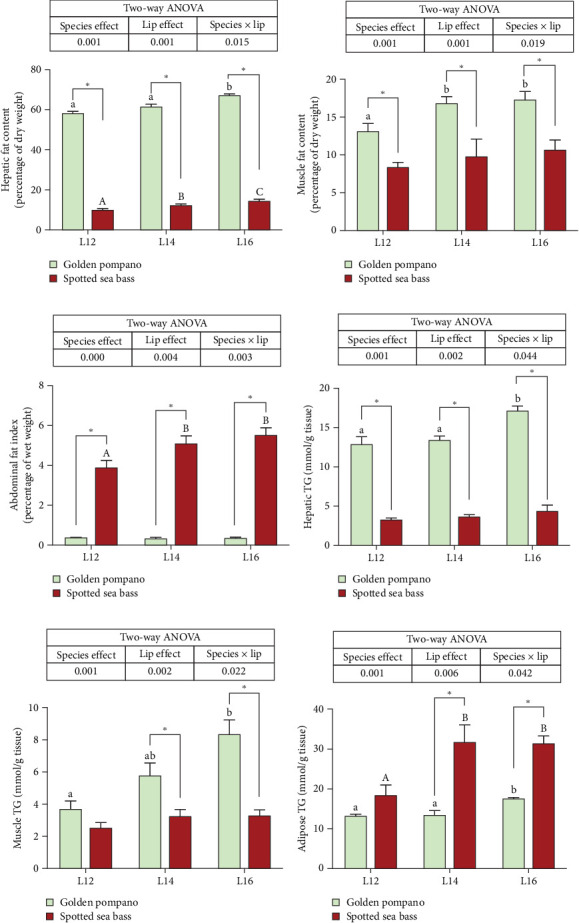
The fat content in liver (a), muscle (b), and abdominal fat index (c) and the triglyceride content in liver (d), muscle (e), and abdominal adipose tissue (f) of golden pompano and spotted sea bass fed the same diets (L12–L16) for 8 weeks. Within-species comparisons: different lowercase letters (a, b, c) denote significant differences among dietary lipid levels for golden pompano, while uppercase letters (a, b, c) indicate differences for spotted sea bass, as determined by one-way ANOVA followed by Tukey's multiple comparison test (*p* < 0.05). Between-species comparisons: asterisks (*⁣*^*∗*^) mark significant differences between golden pompano and spotted sea bass within the same dietary group (*⁣*^*∗*^*p* < 0.05, independent samples *t*-test). Values were analyzed by two-way ANOVA (factors: species × dietary lipid levels). Main effects and interactions with *p* < 0.05 were considered statistically significant. Data are presented as mean ± SEM (*n* = 3 replicates). Abdominal fat index (%) = abdominal fat weight/fish weight × 100.

As shown in Figure 2, two-way ANOVA analysis indicated that crude lipid and TG contents in the liver, muscle, and adipose tissue were significantly influenced by the interaction between dietary lipid levels and fish species (*p* < 0.05). Specifically, crude lipid and TG contents in the liver and muscle of golden pompano were significantly increased with elevated dietary lipid levels, whereas AFI and adipose TG content in spotted sea bass increased in response to higher lipid diets (*p* < 0.05). The golden pompano exhibited higher crude lipid content in both liver and muscle compared to spotted sea bass (*p* < 0.05). Additionally, AFI of golden pompano was markedly lower than that of spotted sea bass (*p* < 0.05). Meanwhile, golden pompano's hepatic TG concentration exceeded that of spotted sea bass by 3.9-fold, with muscular TG levels being 2.5-times higher when both fish were fed diet L16. Conversely, spotted sea bass displayed 1.8-fold greater TG accumulation in abdominal adipose tissue relative to golden pompano (*p* < 0.05). These findings demonstrated that golden pompano preferentially deposits lipids as TG in metabolically active tissues (liver and muscle), while spotted sea bass predominantly stores lipids in specialized adipose depots.

### 3.3. Serum Biochemical Parameters

**Figure 3:**
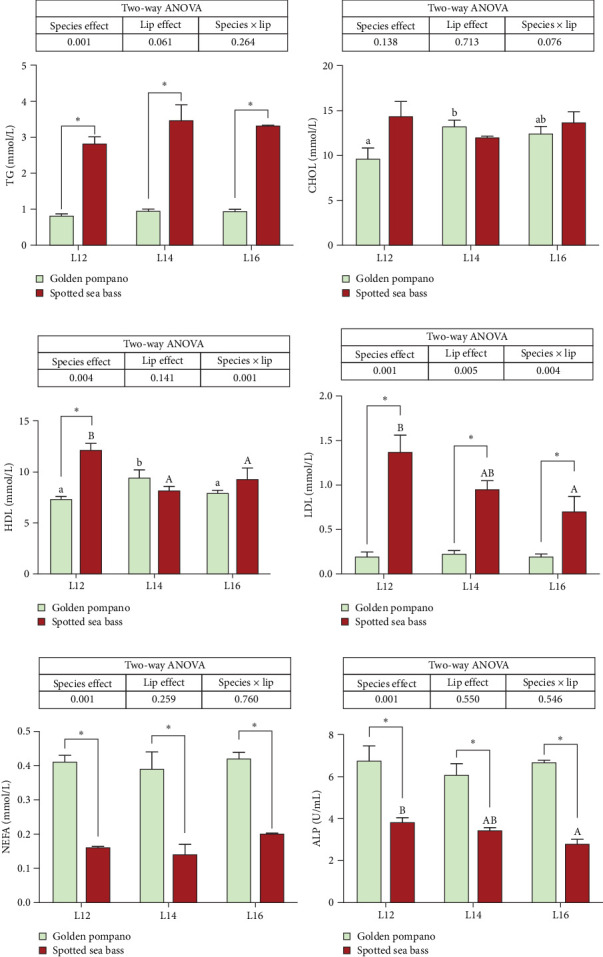
(a–f) Serum triglyceride (TG), cholesterol (CHOL), high-density lipoprotein (HDL), low-density lipoprotein (LDL), nonestesterified fatty acid (NEFA) levels, and alkaline phosphatase (ALP) activities of golden pompano and spotted sea bass fed the same diets (L12–L16) for 8 weeks. Within-species comparisons: different lowercase letters (a, b, c) denote significant differences among dietary lipid levels for golden pompano, while uppercase letters (a, b, c) indicate differences for spotted sea bass, as determined by one-way ANOVA followed by Tukey's multiple comparison test (*p* < 0.05). Between-species comparisons: Asterisks (*⁣*^*∗*^) mark significant differences between golden pompano and spotted sea bass within the same dietary group (*⁣*^*∗*^*p* < 0.05, independent samples *t*-test).Values were analyzed by two-way ANOVA (factors: species × dietary lipid levels). Main effects and interactions with *p*  < 0.05 were considered statistically significant. Data are presented as mean ± SEM (*n* = 3 replicates).

As presented in Figure 3, serum TG and LDL levels were significantly lower in golden pompano than in spotted sea bass across all diets (*p* < 0.05). Serum HDL level was also lower in golden pompano fed the L12 diet. In contrast, serum NEFA and ALP concentrations were higher in golden pompano than in spotted sea bass (*p* < 0.05). Two-way ANOVA analysis showed that HDL and LDL contents were significantly influenced by the interaction between fish species and dietary lipid (*p* < 0.05). No statistically significant differences were detected in CHOL contents between golden pompano and spotted sea bass when fed the same diet (*p* > 0.05).

### 3.4. Transcriptome Analysis

**Figure 4:**
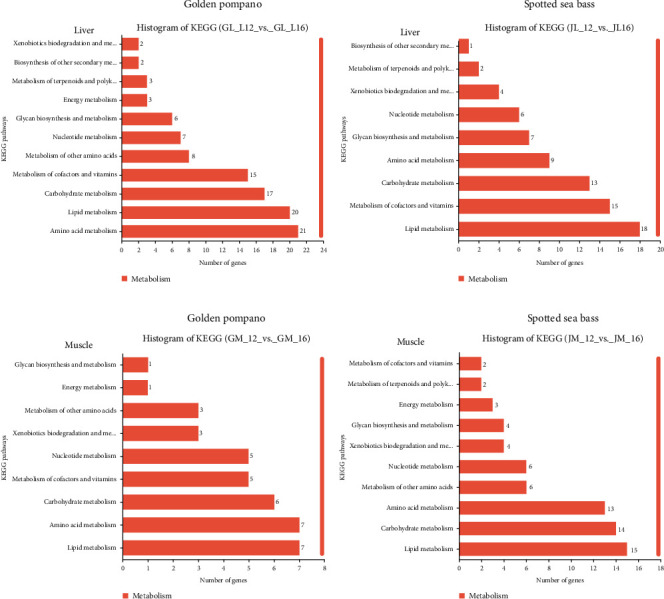
KEGG classification and pathway enrichment of differentially expressed genes (DEGs) in metabolism. (a) the DEGs in the liver of golden pompano between the L12 and L16 groups (GL_L12 VS GL_L16). (b) the DEGs in the liver of spotted sea bass between the L12 and L16 groups (JL_L12 VS JL_L16). (c) the DEGs in the muscle of golden pompano between the L12 and L16 groups (GM_L12 VS GM_L16). (d) the DEGs in the muscle of spotted sea bass between the L12 and L16 groups (JM_L12 VS JM_L16). The ordinate is the name of the KEGG secondary metabolic pathways, the abscissa is the number of DEGs.

The KEGG pathway analysis identified 104, 74, 38, and 69 DEGs assigned to metabolism pathways in the liver and muscle of golden pompano and spotted sea bass, respectively. Among these genes, 20, 18, 7, and 15 DEGs were enriched in the lipid metabolism pathway (Figure 4). Several genes and enriched pathways were identified between L12 and L16 groups in the liver and muscle tissues of golden pompano and spotted sea bass (Tables 3 and 4).

### 3.5. qPCR Validation in the Liver and Muscle

**Figure 5:**
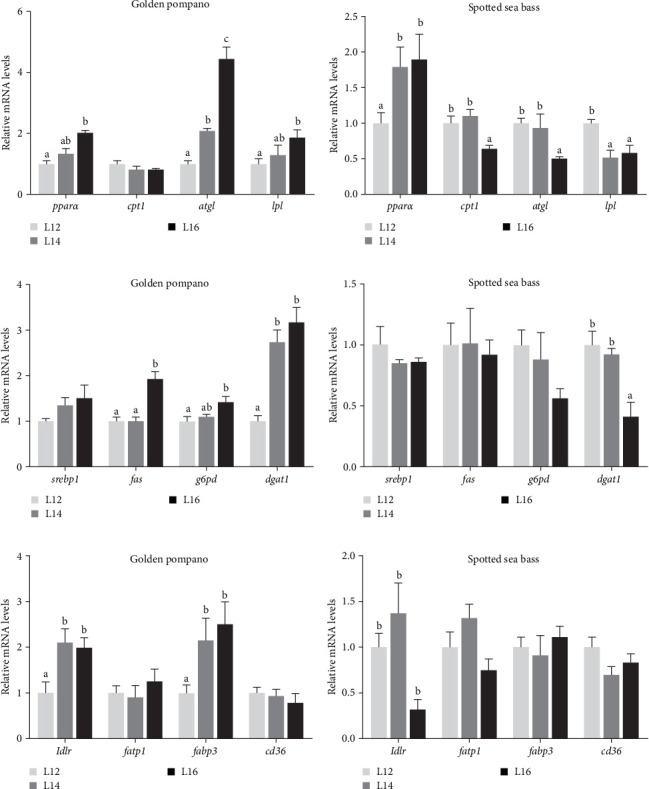
The relative mRNA expression levels of genes involved in fatty acid catabolism, synthesis, and transport in the liver of golden pompano and spotted sea bass fed L12–L16 diets for 8 weeks. (a,b) Lipid catabolism related genes *pparα*, *cpt1*, *atgl*, and *lpl*. (c,d) Lipid synthesis metabolism related genes *srebp1*, *fas*, *g6pd* and *dgat1*. (e,f) Fatty acids transport and uptake related genes *ldlr*, *fatp1*, *fabp3*, and *cd36*. Differences among the L12–L16 groups of each fish species were analyzed using one-way ANOVA followed by Tukey's multiple comparison tests. Bars assigned different superscripts are significantly different (*p* < 0.05).

**Figure 6:**
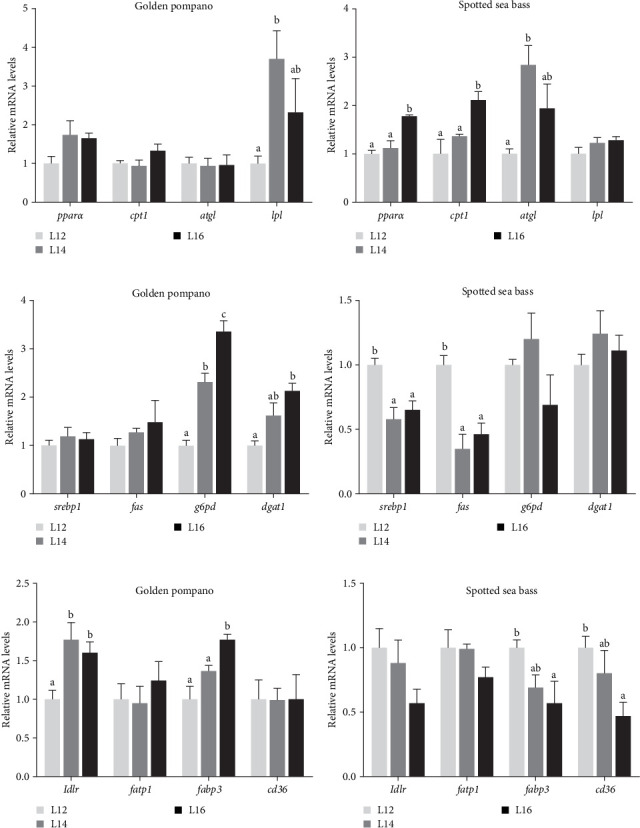
The relative mRNA expression levels of genes involved in fatty acid catabolism, synthesis, and transport in the muscle tissue of golden pompano and spotted sea bass fed L12–L16 diets for 8 weeks. (a,b) lipid catabolism related genes *pparα*, *cpt1*, *atgl*, and *lpl*. (c,d) lipid synthesis metabolism related genes *srebp1*, *fas*, *g6pd*, and *dgat1*. (e,f) fatty acids transport and uptake related genes *ldlr*, *fatp1*, *fabp3*, and *cd36*. Differences among the L12–L16 groups of each fish species were analyzed using one-way ANOVA followed by Tukey's multiple comparison tests. Bars assigned different superscripts are significantly different (*p* < 0.05).

As shown in Figures 5 and 6, several DEGs related to fatty acid β-oxidation, synthesis, and uptake were selected for RT-qPCR validation. Expression patterns were consistent with the RNA-seq results. As depicted in Figure 5, hepatic expression of genes related to lipolysis (*pparα*, *atgl*, and *lpl*), lipid synthesis (*fas* and *g6pd*), fatty acid transport (*ldlr* and *fabp3*), and lipid deposition (*dgat1*) in golden pompano exhibited a significant increase in response to elevated dietary lipid levels (*p* < 0.05). The hepatic expression of genes associated with lipolysis (*cpt1*, *atgl*, and *lpl*) as well as the gene involved in fatty acid transport, *ldlr*, exhibited a significant decrease in spotted sea bass fed L12, L14, and L16 diets (*p* < 0.05). Meanwhile, RT-qPCR analysis on the muscle is shown in Figure 6. A notable upregulation of *g6pd*, *dgat1*, *ldlr*, and *fabp3* gene levels occurred with increasing lipid intake in golden pompano, whereas these genes showed an opposite trend in the muscle of spotted sea bass.

### 3.6. Tissue-Specific Expression of Lipid Metabolism Genes

**Figure 7:**
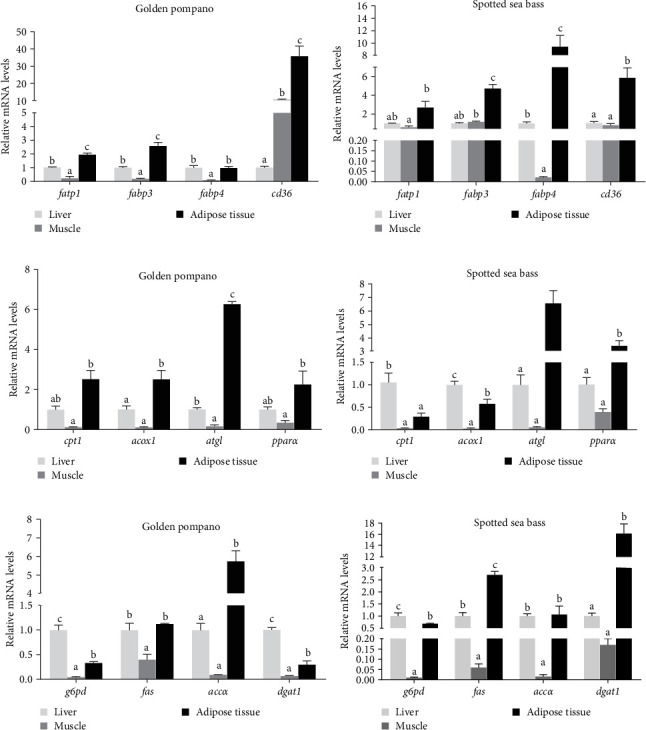
The relative mRNA expression levels of genes involved in fatty acid transport, catabolism and synthesis in the liver, muscle, and abdominal adipose tissue of golden pompano and spotted sea bass fed the L12 diet for 8 weeks. (a,b) fatty acids transport and uptake related genes *ldlr*, *fatp1*, *fabp3*, and *cd36*. (c,d) lipid catabolism related genes *pparα*, *cpt1*, *atgl*, and *acox1*. (e,f) lipid synthesis metabolism related genes *g6pd*, *fas*, *accα*, and *dgat1*. Differential expression of gene related to lipid metabolism in the liver, muscle, and abdominal adipose tissue of each fish species were analyzed using one-way ANOVA followed by Tukey's multiple comparison tests. Bars assigned different superscripts are significantly different (*p* < 0.05).

As shown in Figure 7, fatty acid transport-related genes (*fatp1*, *fabp3*, and *fabp4*) were markedly higher in the liver and abdominal adipose tissue of golden pompano compared to muscle (*p* < 0.05). Conversely, *cd36*-mediated lipid uptake predominated in muscle (*p* < 0.05). Meanwhile, fatty acid catabolism-related genes (*cpt1*, *acox1*, *atgl*, and *pparα*) were predominantly expressed in abdominal adipose tissue versus the liver and muscle, while the *dgat1*-driven reesterification was attenuated in abdominal adipose versus liver and muscle (*p* < 0.05). For spotted sea bass, expression levels of genes associated with fatty acid transport (*fatp1*, *fabp3*, *fabp4*, and *cd36*) exhibited a significant increase in abdominal adipose tissue compared to liver and muscle. Additionally, expression of genes related to fatty acid β-oxidation (*cpt1* and *acox1*) and fat synthesis (*fas* and *dgat1*) was higher in abdominal adipose tissue of spotted sea bass compared with liver and muscle (*p* < 0.05).

### 3.7. Lipid Metabolism and Adipogenesis in Abdominal Adipose Tissue

**Figure 8:**
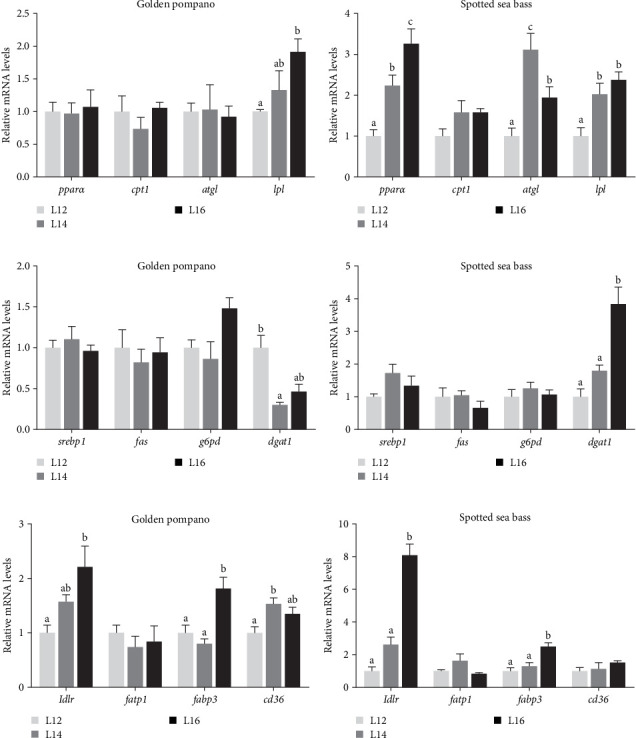
The relative mRNA expression levels of genes involved in fatty acid catabolism, synthesis and transport in abdominal fat tissue of golden pompano and spotted sea bass fed the L12–L16 diets for 8 weeks. (a,b) lipid catabolism related genes *pparα*, *cpt1*, *atgl*, and *lpl*. (c,d) lipid synthesis metabolism related genes *srebp1*, *fas*, *g6pd* and *dgat1*. (e,f) fatty acid transport and uptake related genes *ldlr*, *fatp1*, *fabp3*, and *cd36*. Differences among L12–L16 groups of each fish species were analyzed using one-way ANOVA followed by Tukey's multiple comparison tests. Bars assigned different superscripts are significantly different (*p* < 0.05).

**Figure 9:**
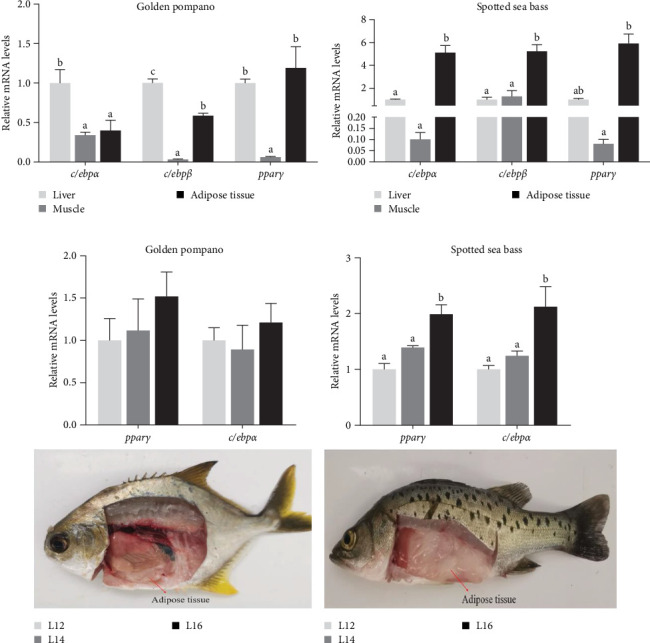
The expression levels of genes involved in adipogenesis in golden pompano and spotted sea bass. The expression of *c/ebpα*, *c/ebpβ*, and *pparγ* in the liver, muscle, and abdominal adipose tissue of golden pompano (a) and spotted sea bass (b) fed the same diet (L12) for 8 weeks. The expression of *pparγ* and *c/ebpα* in the abdominal adipose tissue of golden pompano (c) and spotted sea bass (d) fed L12–L16 diets for 8 weeks. Differential expression of genes related to lipid metabolism in the liver, muscle, and abdominal adipose tissue of each fish species and differences among L12–L16 groups of each fish species, were analyzed using one-way ANOVA followed by Tukey's multiple comparison tests. Columns of each gene without sharing a common letter indicate statistically different at *p* < 0.05. *c/ebpα*, CCAAT/enhancer binding protein alpha; *c/ebpβ*, CCAAT/enhancer binding protein beta; *pparγ*, peroxisome proliferator-activated receptor gamma.

The alterations in gene expression levels associated with lipid metabolism in the abdominal adipose tissue of golden pompano and spotted sea bass, in response to increasing dietary lipid levels, are shown in Figure 8. As dietary lipid levels increased, a significant upregulation in the mRNA expression of *lpl* was observed in the abdominal adipose tissue of both species (*p* < 0.05). In golden pompano, the expression level of *dgat1* in the abdominal adipose tissue showed a significant decrease with increasing dietary lipid levels, whereas spotted sea bass exhibited an opposite trend (*p* < 0.05). Comparative analysis of adipogenesis-related gene expression revealed distinct tissue-specific patterns between the two species. In golden pompano, expression of *c/ebpα* and *c/ebpβ* genes showed a notable increase in liver compared to both muscle and abdominal adipose tissue (Figure 9A). Conversely, spotted sea bass exhibited significantly higher expression of these genes in abdominal adipose tissue relative to liver and muscle (*p* < 0.05) (Figure 9B). While no significant differences in *pparγ* and *c/ebpα* expression were detected among L12–L16 groups in golden pompano (*p* > 0.05), spotted sea bass showed a significant increase in mRNA expression levels of these genes in abdominal adipose tissue with elevation of dietary lipid levels (*p* < 0.05) (Figure 9C, D). These findings collectively indicate that abdominal adipose tissue of spotted sea bass exhibits a higher adipogenic capacity compared to that of golden pompano.

## 4. Discussion

Teleosts exhibit phylogenetically conserved yet species-divergent patterns of lipid partitioning, with preferential deposition in adipose tissue, liver, or muscle [7, 9]. Although excessive dietary lipids can induce metabolic disorders and ectopic fat accumulation [30, 31], our results indicate that species identity rather than dietary lipid level is the primary driver of deposition patterns. This is evidenced by a significant species-by-diet interaction observed for growth performance, morphological indices, and, crucially, fat distribution patterns in a two-way ANOVA. Golden pompano preferentially stored lipids in metabolically active tissues, including liver and muscle, showing significantly higher crude fat and TG concentrations in these sites, whereas spotted sea bass accumulated more fat in specialized adipose depots with higher AFI and adipose TG. Notably, increasing dietary lipid levels exacerbated deposition at these species-specific primary storage sites, consistent with patterns observed in medaka (*Oryzias latipes*) [32], Atlantic salmon (*Salmo salar*) [33], Chinese perch (*Siniperca chuatsi*) [11], and largemouth bass (*Micropterus salmoides*) [34], as well as earlier reports on these two species [9, 35]. Collectively, these results confirm that lipid partitioning in teleosts is governed by inherent species-specific traits, with dietary lipid serving as a modulator that amplifies (rather than reshapes) these evolutionarily conserved deposition preferences—thus clarifying the relative contributions of biological identity and nutritional factors to teleost lipid metabolism.

Transcriptomic and qPCR analyses revealed that these divergent fat distribution patterns are mediated through species-specific metabolic strategies. DEGs enriched in fatty acid transport, synthesis, and degradation pathways indicated that dietary lipid levels modulate lipid deposition via regulation of lipid metabolism genes. In humans, variations in fatty acid uptake across different body regions may contribute to differences in fat distribution [36]. In golden pompano, the upregulation of genes facilitating fatty acid uptake (*lpl*, *fabp3*), de novo lipogenesis (*g6pd*, *fas*), and final esterification (*dgat1*) in liver and muscle indicates a systemic reprograming of these tissues to serve as primary lipid sinks. This is consistent with findings in torafugu, red seabream [37], large yellow croaker [38], and rainbow trout [33]. The mRNA expression level of *dgat1* in the liver and muscle of golden pompano significantly increased with higher dietary lipid levels, while the opposite trend was observed in spotted sea bass. Similar findings were noted in mice, where muscle-specific overexpression of *dgat1* promoted TG deposition [39]. Furthermore, suppression of *dgat1* markedly reduced fat contents induced by lipid overload in hepatocytes of large yellow croaker [40]. These results demonstrate that fat deposition in the liver and muscle of golden pompano increased by increasing fatty acid transport, lipogenesis, and promoting fatty acid reesterified to TG for storage, while the opposite occurred in spotted sea bass, indicating a distinct metabolic strategy.

This species-specificity extends to the expansion capacity of adipose tissue. Usually, the adipose tissue accumulates lipid via two processes: uptake of dietary lipids from circulation and lipogenesis [41]. Here, upregulation of *fabp3* and *ldlr* expression was observed in abdominal adipose tissue of both species in response to increased dietary lipid levels, demonstrating higher fatty acid flux to abdominal adipose tissue. However, their storage efficiency differed markedly. Notably, *dgat1*, a key enzyme catalyzing esterification of fatty acids into TG for tissue storage, exhibited contrasting expression patterns among different species [42, 43]. Expression of *dgat1* in abdominal adipose tissue of golden pompano was lower compared to liver and muscle. Conversely, this pattern was reversed in spotted sea bass, where *dgat1* expression in abdominal adipose was higher than in liver and muscle. These results demonstrate spotted sea bass exhibits a greater rate of fat deposition in abdominal adipose tissue compared to golden pompano. Furthermore, body lipid distribution results from systematic adaptation of lipid metabolism through coordinating different tissues [33]. Normally, TGs are stored in adipose tissue and provide FFAs as a substrate for β-oxidation in peripheral tissues [44]. In this study, spotted sea bass exhibited higher rates of fatty acid uptake and reesterification into TG, leading to predominant lipid deposition in abdominal adipose tissues, with delivery to liver and muscle for fatty acid β-oxidation. Golden pompano's limited adipose *dgat1* activity results in lower fatty acid reesterification locally. Instead, FFAs are diverted to liver and muscle for storage, evidenced by elevated hepatic *dgat1* expression and serum NEFA levels, consistent with patterns observed when adipose storage capacity is exceeded [20, 45, 46]. Thus, we speculated that the differential expandability of abdominal adipose tissue between species is a key determinant of lipid partitioning patterns.

The expansion of adipose tissue can be facilitated by the formation of new adipocytes from precursor cell differentiation during adipogenesis [47]. This process is governed by key transcription factors, including *c/ebps* and *pparγ*, whose activation promotes adipocyte differentiation and lipid storage [48–50]. Here, expression levels of *pparγ*, *c/ebpα*, and *c/ebpβ* were elevated in abdominal adipose tissue of spotted sea bass but decreased in golden pompano. These findings align with results obtained from Nile tilapia, where a high-fat diet induced *pparγ* upregulation and fat deposition [51]. Abdominal adipose tissue of spotted sea bass exhibits higher adipogenic capacity than golden pompano, partly explaining its greater abdominal fat mass. In humans, the activation of *pparγ* in adipose tissue promotes adipocytes differentiation, increasing lipid accumulation in adipose tissue, while decreasing it in liver and muscle [52]. Similarly, *pparγ* and *c/ebpα*-mediated adipogenesis promotes lipid diversion into adipose tissue and away from liver and muscle in spotted sea bass. In contrast, low adipogenic capacity in abdominal adipose of golden pompano allows excessive dietary lipid deposition into liver and muscle through increased fatty acid transport and synthesis. Differences in adipogenesis capacity, fatty acid transport, and synthesis led to distinct body fat distribution patterns.

## 5. Conclusion

In conclusion, this study demonstrates that the adipogenesis of abdominal adipose tissue in spotted sea bass is mediated by *pparγ* and *c/ebpα*, increasing lipid storage capacity and promoting predominant lipid storage in abdominal adipose tissue. In contrast, abdominal adipose tissue of golden pompano has low adipogenesis capacity, while liver and muscle exhibit strong lipid synthesis and fatty acid transport capacities, leading to lipid deposition mostly in these tissues. The capacity for adipogenesis, fatty acid transport, and synthesis contributes to the development of various body fat distribution patterns in fish. Elucidating the mechanism underlying lipid deposition and distribution in different tissues is of great significance for improving growth performance and nutritional quality of fish.

## Figures and Tables

**Table 1:** Ingredients and composition of experimental diets (%).

Ingredients	Diets		
	L12	L14	L16
Fish meal	12	12	12
Poultry meal	15	15	15
Soy protein concentration	10	10	10
Corn gluten meal	14	14	14
Soybean meal	11.9	11.9	11.9
Blend oil^a^	8.2	10.2	12.2
Strong flour	17	17	17
Premix compound^b^	3	3	3
Wheat bran	8.9	6.9	4.9
Total	100	100	100
Proximate composition (%, dry matter basis)			
Dry matter	93.96	90.66	94.36
Crude protein	45.46	45.38	45.32
Crude lipid	12.66	14.4	16.2

^a^Blend oil: consists of fish oil, cotton oil, palm oil, and perilla oil at 3:3:3:1.

^b^Premix compound: consists of 0.5% choline chloride, 0.5% monocalcium phosphate, 1% vitamin mixture, and 1% mineral compound. Vitamin mixture (per kg mixture): VA: 1100000IU; D3:320000IU; VB12:8 mg; VK3:1000 mg; VB1:1500 mg; VB2:2800 mg; VC: 17 mg; VE: 8 mg; calcium pantothenate: 2000 mg; nicotinamide: 7800 mg; folic acid: 400 mg; inositol: 12,800 mg; VB6: 1000 mg. Mineral compound (per kg mixture): sodium fluoride: 2 mg; potassium iodide: 0.8 mg; cobalt chloride (1%): 50 mg; copper sulfate: 10 mg; copper sulfate: 80 mg; zinc sulfate: 50 mg; manganese sulfate: 60 mg; magnesium sulfate: 1200 mg; common salt: 100 mg; zeolite powder: 15.45 g.

**Table 2:** Primer sequence of qPCR for golden pompano and spotted sea bass.

Gene	Golden pompano	Spotted sea bass
*pparα*	F: AATCTCAGCGTGTCGTCTT	F: GACAACGATGCCCTCAGCTC
	R: GGAAATGCTTCGGATACTTG	R: CGGCACAAACTCGACACTCA
*cpt1*	F: CTTTAGCCAAGCCCTTCATC	F: TCCGTGGCAGTCTTCTGAGGTC
	R: CACGGTTACCTGTTCCCTCT	R: GCAGCAGCAGACATACACCTACAG
*atgl*	F: AGCGGCAGGACAGACAAA	F: CTTCCTCTCCGCAACAAGTC
	R: GAGCGAATGAGTGGGAATAAG	R: TGGTGCTGTCTGGAGTGTTC
*lpl*	F: TTTGTCCTTCCTCGTCACCA	F: TGTGTCCAAGTTCTCCCTGCG
	R: AAGACAGCATCCTCTCCACC	R: CCAGCCATGTATCACAATGAAGC
*srebp1*	F: GAGCCAAGACAGAGGAGTGT	F: AGGACACCAAGCCGAATG
	R: GTCCTCTTGTCTCCCAGCTT	R: GTCCTCTTGTCTCCCAGCTT
*fas*	F: GATGGATACAAAGAGCAAGG	F: AGGCATTGTGGAGGGTGTAG
	R: GTGGAGCCGATAAGAAGA	R: CCAGTCCACCAGTGATGATG
*g6pd*	F: GATCTACCGCATAGACCACTACC	F: CCTACATTGCCGAGGACGAA
	R: GAACGACACACGCCACACT	R: ACTGCCAGGGATGTGATGTG
*dgat1*	F: TGGTTTGTTGGTCGCTTCT	F: CGAAGAGCGAGAAAAAGAAGC
	R: GCCTCCTGTCTATAGTGCAGG	R: TGACCACACACCAGTTGAGGA
*ldlr*	F: TACAAATGCGAGTGCGAGG	F: GCCAGTGCAACCATCAATAC
	R: TGACGGTTGGTAAAGAAAAGG	R: CTAAGAGGCTCATCTGACCAA
*fatp1*	F: GGACCCATTACGGCGAT	F: AACCAGCAGGACCCACTACG
	R: CGGTGGTGGAGACATTTTC	R: TCATCCATCACCAACACATCG
*fabp3*	F: TGGGTGTGGGCTTTGCTA	F: TACCAAGCCCACCACTATCATC
	R: CTTAATGGTGCTCTGGGTCTT	R: TCTCGTCAAACTCCTCTCCCA
*cd36*	F: TTCGAGCCAGCCATGTCAGT	F: GTGGGAATAATGGACAGGTGG
	R: ATTGCGTAAGCACCAGCCAC	R: TCTGATGAGAAGAAATAGAGAGGCT
*c/ebpα*	F: AGACCTCGGCGGTGAGA	F: TCTGATTCCAGGGTGTCCTC
	R: CAAGAAGTCGTCGTTGAAAGC	R: TCATCTGCTCAGCCACTCTG
*c/ebpβ*	F: TACCTTCATTACCAGTCCACCAG	F: CACGGACAACGACAGACTGA
	R: CTTCTTTGATTTCCCGCTTG	R: GCCACACACCAACTCACGTA
*pparγ*	F: TCAGGGTTTCACTATGGCGT	F: AGGCCTGCTGAATGTGAAGC
	R: CTGGAAGCGACAGTATTGGC	R: GCTGGATGAAGTGGACGTGG
*β-actin*	F: TACGAGCTGCCTGACGGACA	F: CAACTGGGATGACATGGAGAAG
	R: GCTGTGATCTCCTTCTGC	R: TTGGCTTTGGGGTTCAGG

Abbreviations: *atgl*, adipose triglyceride lipase; *c/ebpα*, CCAAT/enhancer binding protein alpha; *c/ebpβ*, CCAAT/enhancer binding protein beta; *cd36*, cluster of differentiation 36; *cpt1*, carnitine palmitoyltransferase1; *dgat1*, diacylglycerol acyltransferase 1; *fabp3*, fatty acid binding protein3; *fas*, fatty acid synthase; *fatp1*, fatty acid transport protein1; *g6pd*, glucose-6-phosphate dehydrogenase; *ldlr*, low-density lipoprotein receptor; *lpl*, lipoprotein lipase; *pparα*, peroxisome proliferator-activated receptor alpha; *pparγ*, peroxisome proliferator-activated receptor gamma; *srebp1*, sterol regulatory element binding protein 1.

**Table 3:** List of partial genes and enriched pathways related to lipid metabolism in the liver and muscle of golden pompano feeding diets with 12% or 16% lipid for 8 weeks.

Tissue	Pathway/gene	Gene title	log_2_Foldchange	Up/down
Liver L12 vs. L16				
	Map04152 Ampk signaling pathway			
	*Pepck*	Phosphoenolpyruvate carboxy kinase	2.71	Up
	*Creb*	Cyclic AMP-responsive element-binding protein 5	3.14	Up
	*Hsl*	Hormone-sensitive lipase	1.22	Up
	*Hmgcr*	3-hydroxy-3-methylglutaryl-coenzyme A reductase	1.81	Up
	*Hnf4α*	Hepatocyte nuclear factor 4-alpha	−1.29	Down
	Map04979 cholesterol metabolism			
	*Angptl4*	Angiopoietin-related protein 4	1.67	Up
	*Soat*	Sterol O-acyltransferase 1	3.02	Up
	*Lrp1*	Low-density lipoprotein receptor-related protein 1	2.06	Up
	Map00061 fatty acid biosythesis			
	*Acsl*	Long chain fatty acid-CoA ligase 1	1.53	Up
	*Me1*	Malic enzyme	1.15	Up
	Map03320 Ppar signaling pathway			
	*Fabp*	Fatty acid-binding protein	3.16	Up
	*Plin2*	Perilipin-2	1.91	Up
	*Pltp*	Phospholipid transfer protein	2.11	Up
	*FadD*	Long-chain-fatty-acid-CoA ligase 4	3.25	Up
	*Atgl*	Adipose triglyceride lipase	2.29	Up
	Map04975 fat digestion and absorption			
	*Apoe*	Apolipoprotein Eb	−2.4	Down
	*Dgat1*	Diacylglycerol O-acyltransferase 1	2.06	Up
	*Pla2g*	Phospholipase A2	−1	Down
Muscle L12 vs. L16				
	Map04920 adipocytokine signaling pathway			
	*Pepck*	Phosphoenolpyruvate carboxykinase, cytosolic	2.71	Up
	*Glut1/4*	Solute carrier family 2, facilitated glucose transporter member 3	1.39	Up
	Map00071 fatty acid degradation			
	*Cpt1*	Carnitine O-palmitoyltransferase 1	1.15	Up
	*FadD*	Long-chain-fatty-acid-CoA ligase 4	−1.69	Down
	*Acsbg2*	Long-chain-fatty-acid-CoA ligase ACSBG2	−1.26	Down
	Map04975 fat digestion and absorption			
	*ApoCⅠ*	Apolipoprotein C-I	−1.86	Down
	*Pcsk9*	Proprotein convertase subtilisin/kexin type 9	−2.26	Down
	*ApoE*	Apolipoprotein Eb	−2.4	Down
	*Pla2g12*	Group XII secretory phospholipase A2 precursor	−1	Down
	*Dgat1*	Diacylglycerol O-acyltransferase 1	2.06	Up

**Table 4:** List of partial genes and enriched pathways related to lipid metabolism in the liver and muscle of spotted sea bass feeding diets with 12% or 16% lipid for 8 weeks.

Tissue	Pathway/gene	Gene title	log_2_Foldchange	Up/down
Liver L12 vs. L16				
	Map03320 Ppar signaling pathway			
	*Fabp*	Fatty acid-binding protein	−2.33	Down
	*Rxr*	Retinoid X receptor gamma	−1.12	Down
	*Me*	Malic enzyme	−2.41	Down
	*Scd1*	Stearoyl-CoA desaturase 1	−2.73	Down
	*Acsl*	Long-chain acyl-CoA synthetase	−3.21	Down
	Map04152 Ampk signaling pathway			
	*G6pc*	Glucose-6-phosphatase	−1.78	Down
	*Pfk*	ATP-dependent 6-phosphofructokinase	−2.16	Down
	*Creb*	cAMP-responsive element modulator	−3.69	Down
	*Hmgcr*	3-hydroxy-3-methylglutaryl-coenzyme A reductase	−2.06	Down
	*Fas*	Fatty acid synthase	−2.79	Down
	Map04979 cholesterol metabolism			
	*Angptl3/4*	Angiopoietin-related protein 3/4	2.05	Up
	*Lcat*	Phosphatidylcholine-sterol acyltransferase	−1.18	Down
	*Ldlr*	Low-density lipoprotein receptor	−2.67	Down
	*ApoE*	Apolipoprotein Eb	−2.51	Down
	Map01040 biosynthesis of unsaturated fatty acids			
	*Hsd17B12*	Very-long-chain 3-oxoacyl-CoA reductase	−5.55	Down
	*Scd*	Acyl-CoA desaturase	−6.2	Down
	*Acot1*	Acyl-coenzyme A thioesterase 1	2.13	Up
Muscle L12 vs. L16				
	Map04152 Ampk signaling pathway			
	*S6k*	Ribosomal protein S6 kinase	2.02	Up
	*Pgc-1α*	Peroxisome proliferator-activated receptor gamma coactivator 1-alpha	1.48	Up
	*Cpt1α*	Carnitine O-palmitoyltransferase 1	1.14	Up
	*AdipoR*	Adiponectin receptor protein	1.18	Up
	*Igf1r*	Insulin-like growth factor 1 receptor	2.52	Up
	*Mcd*	Malonyl-CoA decarboxylase	1.41	Up
	Map03320 Ppar signaling pathway			
	*Pparα*	Peroxisome proliferator-activated receptor alpha	1.37	Up
	*Hmgcs2*	Hydroxymethylglutaryl-CoA synthase	2.86	Up
	*Lpl*	Lipoprotein lipase	2.26	Up
	*Acsl*	Long-chain-fatty-acid-CoA ligase 1	1.25	Up
	*Pgar*	Angiopoietin-related protein 4	2.33	Up

## Data Availability

The data supporting this study are available from the corresponding author upon reasonable request.
